# Discovery of genomic regions associated with grain yield and agronomic traits in Bi-parental populations of maize (*Zea mays*. L) Under optimum and low nitrogen conditions

**DOI:** 10.3389/fgene.2023.1266402

**Published:** 2023-10-26

**Authors:** Collins Kimutai, Noel Ndlovu, Vijay Chaikam, Berhanu Tadesse Ertiro, Biswanath Das, Yoseph Beyene, Oliver Kiplagat, Charles Spillane, Boddupalli M. Prasanna, Manje Gowda

**Affiliations:** ^1^ Seed, Crop and Horticultural Sciences, School of Agriculture and Biotechnology, University of Eldoret, Eldoret, Kenya; ^2^ International Maize and Wheat Improvement Center (CIMMYT), Nairobi, Kenya; ^3^ Agriculture and Bioeconomy Research Centre, Ryan Institute, University of Galway, Galway, Ireland

**Keywords:** grain yield, low soil nitrogen, maize, sub-Saharan Africa, quantitative trait loci (QTL)

## Abstract

Low soil nitrogen levels, compounded by the high costs associated with nitrogen supplementation through fertilizers, significantly contribute to food insecurity, malnutrition, and rural poverty in maize-dependent smallholder communities of sub-Saharan Africa (SSA). The discovery of genomic regions associated with low nitrogen tolerance in maize can enhance selection efficiency and facilitate the development of improved varieties. To elucidate the genetic architecture of grain yield (GY) and its associated traits (anthesis-silking interval (ASI), anthesis date (AD), plant height (PH), ear position (EPO), and ear height (EH)) under different soil nitrogen regimes, four F_3_ maize populations were evaluated in Kenya and Zimbabwe. GY and all the traits evaluated showed significant genotypic variance and moderate heritability under both optimum and low nitrogen stress conditions. A total of 91 quantitative trait loci (QTL) related to GY (11) and other secondary traits (AD (26), PH (19), EH (24), EPO (7) and ASI (4)) were detected. Under low soil nitrogen conditions, PH and ASI had the highest number of QTLs. Furthermore, some common QTLs were identified between secondary traits under both nitrogen regimes. These QTLs are of significant value for further validation and possible rapid introgression into maize populations using marker-assisted selection. Identification of many QTL with minor effects indicates genomic selection (GS) is more appropriate for their improvement. Genomic prediction within each population revealed low to moderately high accuracy under optimum and low soil N stress management. However, the accuracies were higher for GY, PH and EH under optimum compared to low soil N stress. Our findings indicate that genetic gain can be improved in maize breeding for low N stress tolerance by using GS.

## 1 Introduction

Maize (*Zea mays* L.), a staple crop in sub-Saharan Africa (SSA), is projected to increase in demand (Ekpa et al., 2018), necessitating improved breeding systems to maximize productivity. Over the years, maize productivity in SSA has been limited by the adverse effects of parasitic weeds (e.g., *Striga sp*. (Kanampiu et al., 2018; Yacoubou et al., 2021)), insect infestations (e.g., fall armyworm (Baudron et al., 2019; De Groote et al., 2020)), and disease incidences (e.g., Maize lethal necrosis (Boddupalli et al., 2020)). Most notably, inadequate soil fertility (particularly low nitrogen) is a primary cause of low maize yields in the region’s smallholder farming communities. Modelling studies have established that climate change has the potential to worsen these current maize production constraints (Tesfaye et al., 2015), especially under low soil nitrogen conditions (Falconnier et al., 2020). In this regard, maize breeding holds a significant potential to provide sustainable solutions to the prevailing and projected biotic and abiotic constraints (Eriksson et al., 2018; Ndlovu, 2020).

As nitrogen use efficiency (NUE) is critical for sustainable productivity, there is major interest in developing varieties that perform better in low soil nitrogen stress conditions (Ribaut et al., 2007). However, to harness plant breeding for such stress conditions, an understanding of the performance of maize genotypes under low nitrogen conditions is required. Assessing the performance of maize genotypes under different nitrogen regimes is critical for identifying promising parental lines. Phenotypic evaluations can reveal useful diversity in maize germplasm (Shitta et al., 2021; Chen et al., 2023a). However, morphological characterizations of genotypes are influenced by both the genotype (G) and environment (E), as well as G × E interaction, and hence may not accurately reflect the genotypic effect of nitrogen utilization *per se* (de Carvalho et al., 2012). In addition, large genotype X season and genotype × location interactions can stymie progress in selecting for low nitrogen tolerance (Ribaut et al., 2007). In this context, understanding the genetic architecture of grain yield (GY) and other secondary traits that are associated with low soil nitrogen can speed up genetic improvement (Ertiro et al., 2020c) for low nitrogen tolerance in tropical maize.

Maize GY under low soil nitrogen is a complex trait governed by multiple genes. In most cases, maize breeding for low nitrogen focuses on the anthesis-silking interval (ASI), anthesis date (AD), plant height (PH), ear position (EPO), and ear height (EH) for indirect selection and GY for direct selection (Worku et al., 2007; Worku et al., 2008; Worku et al., 2012; Das et al., 2019; Ertiro et al., 2020b; Ertiro et al., 2022; Ndlovu et al., 2022). However, morphological characterizations are time-consuming, cost-ineffective, and lack accuracy. The complex nature of breeding maize under low nitrogen conditions has necessitated the use of quantitative trait loci (QTL) based approaches which have been shown to contribute significantly to the understanding of the genetic basis of crop performance and stability under nitrogen-stressed conditions (Semagn et al., 2015). The discovery and characterization of QTL can assist breeders in using genomic regions linked with complex trait expression and deciphering their genetic contribution at the target loci (Ertiro et al., 2020c). In addition, through QTL analysis, the biological mechanisms responsible for phenotypic expression can be pursued. Furthermore, exploiting molecular markers in breeding has allowed the mapping of a subset of markers associated with one or more QTL that contribute to regulating the expression of complex traits like GY. Such molecular markers can form the concrete basis for the use of other genomic approaches such as genomic selection in the desired population (Würschum, 2012). Unfortunately, little is known about the genetic basis of GY and associated traits under low nitrogen conditions, and major effect QTLs are yet to be reported (Ertiro et al., 2020c).

Genomic selection (GS) is a tool increasingly used to reduce the breeding cycle length and increase genetic gain in both crops and livestock (Meuwissen et al., 2001). The success of GS in the dairy industry (where the breeding cycle of the cattle can be reduced from 7 years to 18 months) has helped to achieve twice the genetic gain for key traits (García-Ruiz et al., 2016). Such successes have motivated plant breeders to apply GS in crop improvement for reducing the breeding cycle and increase genetic gain. Currently, GS is applied to improve a range of different traits in maize (Beyene et al., 2015; Ertiro et al., 2020a; Kibe et al., 2020; Gowda et al., 2021; Ndlovu et al., 2022), wheat (Dreisigacker et al., 2021; Bonnett et al., 2022; Juliana et al., 2022; Ficht et al., 2023), and other crops (Hu et al., 2022; Qin et al., 2022; Chen et al., 2023b). GS has been successfully integrated into maize breeding programs for improving GY under optimum and drought conditions (Beyene et al., 2015; Zhang et al., 2017b; Vivek et al., 2017; Beyene et al., 2019; Wang et al., 2020; Beyene et al., 2021). Linkage mapping enables the detection of QTL for the target trait by using different bi-parental populations, whereas GS enables the selection of superior individuals by considering the effects of multiple genes controlling a target trait (Crossa et al., 2017; Yuan et al., 2019). Combining linkage mapping results with GS will accelerate the breeding efficiency for GY and other complex traits under low N stress conditions. Therefore, the full potential of GS needs to be assessed in biparental and/or practical breeding populations for low soil N tolerance.

Studies conducted over the last 20 years have identified QTL that generally explained a significant portion of the phenotypic variance, and therefore gave rise to an optimistic assessment of the prospects of marker-assisted selection (Semagn et al., 2010). According to Coque and Gallais (2006), the detected marker QTL associations in maize revealed the consistency of the involvement of some traits, such as root architecture and glutamine synthase activity, which would be of major importance for GY setting under both optimum and low nitrogen conditions. The discovery of genomic regions associated with GY and other agronomic traits in maize under low soil nitrogen conditions is of paramount importance. Hence, this study sought to evaluate four F3 populations to 1) estimate the phenotypic effect and heritability for GY and other agronomic traits under optimum and low nitrogen management 2) identify the genomic regions associated with these traits, and 3) assessing the usefulness of GS in improving GY and other agronomic traits under optimum and low soil N conditions. The findings of this study can provide genetic resources that can be used in scaling the application of MAS for enhancing maize GY in nitrogen-starved soils in SSA. Furthermore, the identification of QTL can hasten maize breeding cycles and facilitate the release of nitrogen-tolerant or NUE varieties for resource-constrained smallholder farmer communities in the region.

## 2 Materials and methods

### 2.1 Plant materials

Four F3 tropical maize populations were evaluated in Kenya and Zimbabwe. The populations were developed by the Global Maize Program of the International Maize and Wheat Improvement Center (CIMMYT). The specific details of the tested populations are presented in Table 1. These lines are adapted to mid-altitude regions (1,000–1500 M Above sea level) of SSA. They are bred on a good-by-good basis and adapted to stress conditions. Populations are test crossed with an appropriate tester from the opposite heterotic group for phenotypic evaluations. The CIMMYT lines utilized for the project were derived from breeding programs targeting tolerance to low soil nitrogen; hence the best choice for QTL mapping with most of the lines included in two association mapping panel (AMP) constituted under IMAS (improved maize for Africa soils) and DTMA (Drought Tolerant Maize for Africa) projects (Semagn et al., 2012; Ertiro et al., 2020a). The four populations formed a set of multiple bi-parental populations used in the current study.

**TABLE 1:** Details of the populations used in this study and number of locations evaluated under optimum and low N management.

Population	Pedigree	Tester	Population size	No of locations	
				Opt	Low N
**POPULATION 1**	CML494×CML550	CML495	357	3	4
**POPULATION 2**	CKL05017× CML536	CML444× CML395	276	3	3
**POPULATION 3**	CML550× CML507	CML442× CML312	315	2	1
**POPULATION 4**	VL081452× VL058589	CML444× CML395	158	1	2

*Opt, Optimum; Low N–Low soil N management.

### 2.2 Field trial

All four populations were evaluated for response to low soil nitrogen conditions at one to four different locations; Kiboko (Longitude 37°E, Latitude 2°S, 975 M A.S.L), Embu (Longitude 37°E, Latitude 3°S, 1560 M A.S.L), and Harare in Zimbabwe (Longitude 31^0^ E, Latitude 17°S, 1490 M A.S.L). The lines were evaluated on an alpha lattice incomplete block design under two nitrogen levels. The two nitrogen treatments were low N (N-depleted field/plot with no application of N fertilizer) and at normal farmer practice conditions (optimum N 200 kg/ha). The low-N sites were prepared by depleting N by growing sorghum at high density with no N fertilizer sources added for four cropping cycles. The depletion crop was uprooted at near maturity and removed from the low-N field to prevent the incorporation of crop residues into the soil. A soil nitrate concentration ranging from 7.5 to 15 parts per million (ppm) is indicative of soil nitrogen deficiency (Zaman-Allah et al., 2018). Low N sites with around 10 ppm of nitrate level provide good experimental conditions for detecting useful genetic variation. The sites used in the present study showed soil nitrate levels ranging from 0.10 to 0.12 ppm in stress experimental sites, while in the optimum sites, the levels were greater than 0.25 ppm. Single row plots measuring 5 m long at 0.75 m row spacing with two seeds per hill were sown. After 3 weeks of planting, plants were thinned to one plant per hill to obtain a final population density of 53,333 plants per hectare. All entries were planted on the same day in conventionally tilled plots and maintained under rain-fed or irrigated conditions.

### 2.3 Data collection

#### 2.3.1 Phenotyping of important agronomic traits

Ten plants in the middle of the row were selected for each genotype for phenotypic evaluation. Phenotypic components measured and analyzed are plant height (PH in centimeters), anthesis date (AD, days), anthesis silking interval (ASI, days), ear height (EH, in centimeters), ear position (EPO, ratio of EH/PH), number of ears per plant (EPP, total number of ears per plot divided by number of plants per plot) and grain yield (GY in t/ha). Mature ears were harvested, manually bagged, air-dried, and shelled on an electric shelling device. The total GY of each plot was weighed on a balance and converted to GY into t/ha.

Best linear unbiased predictors (BLUPs) were calculated with mixed model where genotypes and other factors were treated as random except replications and best linear unbiased estimators (BLUEs) were calculated where genotype entries and replications are treated as fixed effects and the rest of the terms as random. Estimating broad-sense heritability, all the terms were considered random. Broad sense heritability was estimated by the formula:

$$h^{2}=\sigma_{G}^{2}/\left(\sigma_{G}^{2}+\sigma_{\text{GE}}^{2}/E+\sigma_{e}^{2}/\text{Er}\right)$$

Where **σ** ^**2**^_**G**_ is the genotypic variance, **σ** ^**2**^_**GE**_ is the Genotypic by environment interaction (GEI), **σ** ^**2**^_**e**_ is the error variance, **E** is the number of environments and **r** is the number of replications in each trial. The phenotypic and genotypic correlations among traits were evaluated as described by Hirel et al. (2007).

### 2.4 Genotypic analysis

Phenotypic data was collected on testcross hybrids whereas F_3_ population lines were genotyped. Since, the single tester was used for each population, assumption is tester effect is same across lines, therefore, tester effect was not included in the model for analyses. Total DNA was extracted from bulked young leaves of the lines according to the CTAB method (CIMMYT, 2005), and the DNA quality for each sample was checked using gel-electrophoresis and spectrophotometer (NanoDrop ND8000 Thermo Scientific) before genotyping. Genotyping was performed using the Illumina MaizeSNP1500 Bead Chip evenly spaced SNP to cover the whole maize genome (Ganal et al., 2011). The above task was performed at LGC genomic labs in the United Kingdom (https://www.lgcgroup.com/genotyping/).

Markers which are homozygous for both the parents and polymorphic between them were retained for mapping in each population. Linkage maps in all four populations were constructed using QTL IciMapping version 4.1 software (Meng et al., 2015). Finally, we used 202, 452, 384, and 387 high-quality SNPs in F_3_pop1, F_3_pop2, F_3_pop3, and F_3_pop4, respectively. Linkage map was constructed by using these SNPs, by selecting the most significant markers using stepwise regression. A likelihood ratio test was used to calculate the logarithm of odds (LOD) for each marker at score of >3 with a 30 cM maximum distance between two loci. The recombination frequency between linked loci was transformed into cM (centi Morgan) using Kosambi”s mapping function (Kosambi, 1916). BLUPs across environments were used to detect QTLs based on inclusive composite interval mapping (ICIM) for each population. Phenotypic variation explained by individual QTL and total variation explained by all QTLs together was estimated. QTL naming was done with letter “q” indicating QTL, followed by abbreviation of trait name, the chromosome and marker position, respectively (Ribaut et al., 1997). Additive (a) and dominance (d) effects for each QTL as estimated with QTL IciMapping v.4.1 were used to calculate the ratio of dominance level (|d/a|). This ratio was used to classify the nature of QTL: additive (A; 0 ≤ |d/a| ≤ 0.2); partially dominant (PD; 0.2 < |d/a| ≤ 0.8); dominant (D; 0.8 < |d/a| ≤ 1.2) and overdominant (OD; |d/a| > 1.2).

### 2.5 Genomic prediction

Genomic prediction (GP) analyses were conducted in R program version 4.2.1 (R Core Team 2023). GP was applied on each F_3_ population to find out the prediction ability of GY and other agronomic traits evaluated in optimum and low soil N conditions. We used GP model RR-BLUP to predict the untested lines using a five-fold cross validation (Zhao et al., 2012; Crossa et al., 2017). BLUEs across environments for each of the F_3_ population were used for the analysis. For GS analyses polymorphic SNPs between the parents of each population was used, like 202, 452, 384, and 387 SNPs in F_3_pop1, F_3_pop2, F_3_pop3 and F_3_pop4, respectively were used. We applied five-fold cross validations with ‘within population’ approach where both training and estimation set are derived from within each of biparental population. For each trait in each population, 100 iterations were done for sampling of the training and estimation sets. The prediction accuracy was calculated as the correlation between the observed and predicted breeding values divided by the square root of heritability (Dekkers, 2007).

## 3 Results

### 3.1 Phenotypic distributions and correlation of traits

**FIGURE 1:**
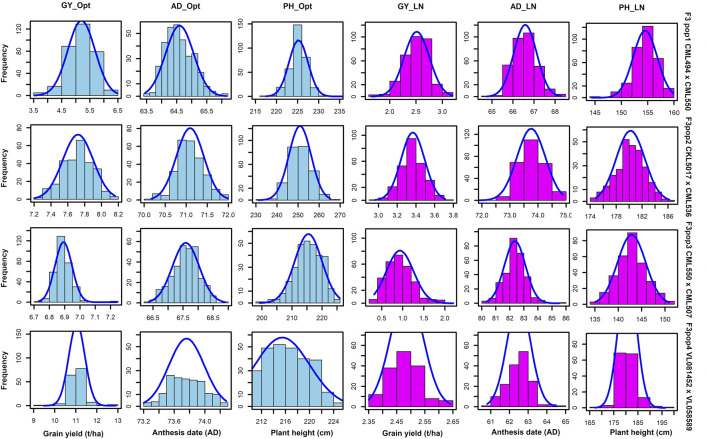
Phenotypic distribution for GY, AD and PH under optimum and low soil nitrogen conditions. The sky blue and pink colour denotes trials conducted under optimum and low nitrogen conditions, respectively. GY–Grain yield; AD - anthesis date; PH–plant height.

**FIGURE 2:**
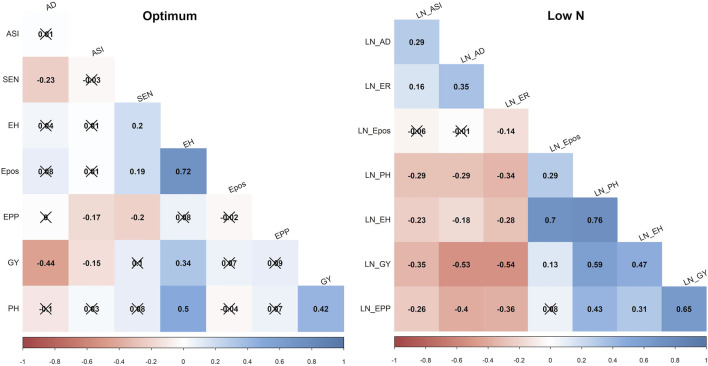
Phenotypic correlations of GY and other agronomic traits evaluated under optimum and low soil nitrogen conditions in F_3_ pop1 CML494 x CML550 population. The x marks indicated values are not significant at *p* < 0.05. LN–low soil N conditions. Correlations with >0.11, and >0.19 were significant at 0.05, and 0.01 (p) levels, respectively. GY–Grain yield; AD - anthesis date; ASI - anthesis silking interval; PH–plant height; EH, ear height; Epos–ear position; EPP–ears per plant; SEN–senescence.

GY, ASI, AD, PH, EPO, and EH varied widely across the two nitrogen regimes (i.e., optimum and low nitrogen environments). The extent of variation, however, differed between the four bi-parental maize populations. Low nitrogen conditions increased the trial mean for AD and decreased the trial mean for GY and PH across the four F_3_ populations tested in this study (Figure 1, Supplementary Table S1). GY showed a negative genotypic correlation with AD and ASI across the two nitrogen regimes (Figure 2). Moreover, consistent positive genetic correlations for GY were recorded with EH, EPO, and PH.

### 3.2 Trial mean, genetic variance, and heritability of traits

Population 1 (CML494xCML550): Across the study sites, average GY was 5.22 t/ha and 2.52 t/ha under optimum and low soil nitrogen conditions, respectively (Table 2). A margin of 2-day extension in AD was observed under low soil nitrogen conditions. The genotypic and genotype × environment interaction effects were significant (*p* ≤ 0.05) for GY. Under optimum conditions, moderate heritabilities across environments were recorded for AD (0.55), GY (0.54), and EH (0.51). Similarly, AD (0.62), GY (0.58), and EH (0.55) exhibited slightly higher heritability under low nitrogen conditions. The lowest heritabilities under optimum nitrogen conditions were exhibited by PH (0.36) and EPO (0.42) traits. ASI (0.18), PH (0.39), and EPO (0.48) recorded the lowest heritability values under low nitrogen conditions.

**TABLE 2:** Trait means, heritability and variance components of GY and other traits evaluated under optimum and low nitrogen conditions in four F_3_ populations across multiple environments.

	Trait	Mean	σ^2^_G_	σ^2^_GxE_	σ^2^_e_	H^2^
**F3 pop1 CML494**×**CML550**						
**Optimum**	GY	5.22	0.44**	0.51**	1.25	0.54
	AD	64.58	0.42**	0.26**	1.53	0.55
	PH	225.27	17.10**	12.19**	161.99	0.36
	EH	107.94	15.09**	1.23**	82.84	0.51
	EPO	0.46	0.0002*	0.0000	0.0012	0.42
**Low N**	GY	2.52	0.11**	0.10**	0.41	0.58
	AD	66.57	0.53**	0.30**	1.97	0.62
	ASI	−0.51	0.08*	0.06*	1.35	0.18
	PH	154.64	14.92**	23.10**	137.33	0.39
	EH	67.05	11.84**	7.55**	62.73	0.55
	EPO	0.41	0.0002*	0.00	0.002	0.48
**F3 pop 2 CKL5017**×**CML536**						
**Optimum**	GY	7.72	0.12**	0.15**	1.60	0.30
	AD	71.10	0.42**	0.03**	2.34	0.41
	PH	250.79	45.01**	4.99**	74.41	0.68
	EH	83.98	33.01**	0.00	129.88	0.68
	EPO	0.46	0.0003**	0.00	0.003	0.41
**Low N**	GY	3.37	0.06**	0.01*	0.84	0.31
	AD	73.75	0.70**	0.29**	3.49	0.41
	PH	180.17	16.61**	5.91**	185.53	0.34
	EH	83.98	20.01**	0.00	126.88	0.49
	EPO	0.46	0.0003*	0.00	0.004	0.41
**F3 pop 3 CML550**×**CML507**						
**Optimum**	GY	6.89	0.11*	0.14**	1.24	0.31
	AD	67.59	0.50**	0.16**	2.32	0.43
	PH	215.46	43.72**	4.35**	156.63	0.51
	EH	106.11	14.93**	4.33**	94.40	0.37
**Low N**	GY	0.94	0.03*	-	0.13	0.29
	AD	82.37	1.10**	-	1.69	0.57
	PH	142.88	26.73**	-	83.97	0.39
	EH	63.55	10.25**	-	34.65	0.37
**F3 pop 4 VL081452**×**VL058589**						
**Optimum**	GY	11.05	0.53**	-	2.82	0.27
	AD	73.76	0.32*	-	2.86	0.18
	PH	262.81	12.96**	-	194.11	0.14
	EH	2.01	1.08**	-	7.08	0.20
**Low N**	GY	2.49	0.08*	0.05*	0.42	0.39
	AD	62.49	0.70**	0.27**	1.47	0.58
	ASI	1.27	0.26**	0.08**	3.17	0.21
	PH	173.09	40.44**	0.00	93.40	0.63

*, ** Significant at *p*< 0.05 and *p*< 0.01 levels, respectively. Low N–low soil N conditions. GY, Grain yield; AD, anthesis date; ASI, anthesis silking interval; PH, plant height; EH, ear height; EPO, ear position; σ^2^_G,_- genotypic variance; σ^2^_GxE,_- genotypic × environment interaction variance; σ^2^_e,_- residual error variance; H^2^ - heritability.

Population 2 (CKL5017xCML536): Under optimum conditions (Table 2), moderate heritability was observed for PH (0.68) and EH (0.49) whilst GY (0.28) recorded low heritability across the studied sites. Under low nitrogen conditions, all the studied traits recorded low heritabilities with GY pegged at 0.31. The genotypic and genotype × environment interaction effects were significant (*p* ≤ 0.05) for GY. Across the studied regions, the mean GY was 7.72 t/ha and 3.37 t/ha under optimum and low nitrogen conditions, respectively.

Population 3 (CML550xCML507): Low heritabilities were recorded across the tested traits under both optimum and low nitrogen conditions (Table 2). GY heritabilities were 0.31 and 0.29 under optimum and low nitrogen conditions, respectively. The genotypic and genotype × environment interaction effects were significant (*p* ≤ 0.05) for GY. In this population, the mean GY was 6.89 t/ha and 0.94 t/ha under optimum and low nitrogen conditions, respectively.

Population 4 (VL081452xVL058589): All traits tested using population 4 under optimum conditions recorded low heritabilities (Table 2) including GY (0.2*7*). Under low nitrogen conditions, AD (0.58) exhibited moderate heritability whilst GY (0.39) and ASI (0.21) had the lowest heritabilities. The genotypic and genotype × environment interaction effects were significant (*p* ≤ 0.05) for GY. Across the study sites, average yields were 11.05 t/ha and 2.49 t/ha under optimum and low nitrogen conditions, respectively.

### 3.3 QTLs mapping in four F_3_ maize populations

The study obtained a total of 906.83, 1,650.28, 1857.12, and 2169.97 cM from 202, 452, 384, and 387 polymorphic SNPs for F_3_ population 1, 2, 3, and 4, respectively (Table 3). The mean distance between adjacent markers was lowest in population 2 (3.65 cM) and highest for population 4 (5.61 cM). Across the ten maize chromosomes, a total of 91 significant QTLs were identified for GY (11), AD (26), PH (19), ASI (4), EH (24), and EPO (7) under optimum (52) and low nitrogen (39) conditions (Tables 4–7). The identified QTLs were distributed across 10 chromosomes. Across environments, chromosomes 1 (16) and 8 (14) had the highest number of QTLs, whilst chromosomes 7 (5) and 5 (6) had the lowest number of QTLs. The identified QTLs were distributed as 19, 36, 18, and 36 for populations 1, 2, 3, and 4, respectively. Proportional phenotypic variance for each QTL ranged from 0.7% (AD for population 2 under optimum nitrogen) to 15.22% (AD for population 4 under low nitrogen).

**TABLE 3:** Linkage map information for the four F3 populations used for QTL analysis.

Chromosome	F3 pop1 CML494×CML550		F3 pop 2 CKL5017×CML536		F3 pop 3 CML550×CML507		F3 pop 4 VL081452×VL058589	
	SNPs	Distance (cM)	SNPs	Distance (cM)	SNPs	Distance (cM)	SNPs	Distance (cM)
1	28	114.37	73	304	63	279.88	74	392.13
2	15	101.71	57	234	42	234.4	39	207.9
3	33	122.62	45	180.5	43	232.17	33	235.77
4	26	83.39	55	195.97	35	220.4	55	235.5
5	25	125.19	53	137.22	54	213.61	50	225.05
6	17	101.46	32	121.78	33	148.3	33	243.07
7	20	47	30	91.55	23	71.67	27	86.99
8	18	55.6	46	172.03	36	108.61	30	174.8
9	13	80.04	27	115.8	23	151.78	20	230.13
10	7	75.45	34	97.43	32	196.3	26	138.33
Total	202	906.83	452	1,650.28	384	1857.12	387	2169.67
Avg dist		4.49		3.65		4.84		5.61

**TABLE 4:** Genetic characteristics of QTLs detected for GY and associated traits under optimum and low-nitrogen stress in F_3_ population 1.

Mgmt	Trait	Chr	QTL name	Position (cM)	Left marker	Right marker	Physical position (Mbp)	LOD	PVE (%)	TPVE (%)	Add	Dom	QTL
**F3 pop1 CML494**×**CML550**													
Opt	GY	1	*qGY01_21*	83	PZA02487.1	PZA00425.11	20.99–21.87	4.94	6.03	11.58	−0.18	0.009	A
		9	*qGY09_30*	60	PZB01110.6	PHM13183.12	29.66–76.42	5.39	6.91		0.19	0.029	A
	AD	1	*qAD01_246*	27	PZB00895.1	PZA01588.1	245.60–257.19	3.45	5.17	8.09	−0.14	−0.063	PD
	PH	7	*qPH07_88*	13	PZB00174.1	PZA01690.7	87.78–105.88	3.32	6.00	6.00	−2.20	−1.069	PD
	EH	1	*qEH01_21*	83	PZA02487.1	PZA00425.11	20.99–21.87	3.42	5.07	23.20	−0.71	−0.071	A
		4	*qEH04_115*	46	PZA00704.1	PZA03564.1	114.90–119.66	3.53	5.30		−0.72	−0.039	A
		8	*qEH08_60*	23	PZA02683.1	PZA00379.2	59.15–74.72	5.42	8.46		−0.87	0.216	PD
		10	*qEH10_95*	75	PHM229.15	PZB01358.1	92.58–107.42	3.40	5.22		0.74	−0.037	A
	EPO	6	*qEP06_157*	0	PHM5361.13	PZA00889.2	156.17–159.55	4.68	6.16	15.56	0.00	0.002	PD
		8	*qEP08_60*	23	PZA02683.1	PZA00379.2	59.15–74.72	9.39	12.76		0.00	0.003	PD
Low N	AD	8	*qAD08_128*	15	PZB01454.1	PHM15744.10	127.95–138.96	3.05	5.00	7.70	−0.16	0.023	A
	ASI	1	*qASI01_21*	96	PZA00425.11	PZA00566.5	9.53–20.99	3.81	3.96	6.91	0.03	−0.045	OD
		3	*qASI03_171*	88	PHM17210.5	PZA00538.15	170.91–199.68	3.02	3.07		0.03	0.027	D
		6	*qASI06_71*	83	PZA02606.1	PZA01960.1	70.29–156.68	3.03	6.80		0.02	0.075	OD
	PH	1	*qPH01_22*	73	PZA01963.15	PZA02487.1	21.86–191.32	4.46	9.01	9.11	−1.06	0.692	PD
		6	*qPH06_120*	40	PZA02673.1	PZA00473.5	117.06–135.75	3.50	3.19		−0.53	0.633	D
		7	*qPH07_120*	22	PZA01542.1	PZA02854.13	112.40–120.22	4.13	3.76		−0.74	−0.146	A
	EH	7	*qEH07_106*	20	PZA01690.7	PZA01542.1	105.88–112.40	3.50	4.87	4.87	−0.72	−0.222	PD
	EPO	4	*qEP04_115*	46	PZA00704.1	PZA03564.1	114.90–119.66	3.08	3.80	11.56	−0.01	−0.003	PD
		6	*qEP06_157*	1	PHM5361.13	PZA00889.2	156.17–159.55	5.82	7.76		0.01	0.006	D

Opt, Optimum; Low N- low soil N stress management; GY, Grain yield; AD, anthesis date; ASI, anthesis silking interval; PH, plant height; EH, ear height; EPO, ear position. The italic values refer to the names of identified QTLs.

**TABLE 5:** Genetic characteristics of detected QTLs for GY and associated traits under optimum and low-nitrogen stress in F3 population 2.

Mgmt	Trait	Chr	QTL name	Position (cM)	Left marker	Right marker	Physical position (Mbp)	LOD	PVE (%)	TPVE (%)	Add	Dom	QTL
**F3 pop 2 CKL5017**×**CML536**													
Opt	GY	3	*qGY03_10*	144	PZD00038.2	PHM4621.57	4.65–159.08	3.41	4.08	16.52	−0.15	−0.047	PD
		8	*qGY08_127*	100	PZA00460.8	PHM4203.11	126.42–156.31	4.84	4.15		0.15	0.059	PD
	AD	1	*qAD01_215*	188	PHM759.24	PZA00664.3	213.95–216.05	4.96	0.70	18.89	0.40	0.002	A
		2	*qAD02_55*	95	PHM3055.9	PHM4259.5	51.96–186.31	9.84	2.20		−4.19	−4.208	D
		2	*qAD02_145*	151	PHM5060.12	PZA03211.6	142.76–184.40	17.23	2.23		−0.06	8.428	OD
		3	*qAD03_158*	180	PZA00186.4	PZA01396.1	157.97–159.08	14.47	2.23		4.20	−3.541	D
		4	*qAD04_14*	50	PHM16788.6	PHM 2006.57	13.89–171.71	9.06	2.20		4.14	−4.264	D
		5	*qAD05_10*	18	PZA00963.3	PZA00818.1	1.1–208.34	14.45	2.23		−4.19	−4.629	D
		6	*qAD06_72*	1	PHM2898.24	PZD00072.2	71.99–72.48	14.32	2.21		4.20	−3.821	D
		9	*qAD09_23*	114	PHM4720.12	PZA03671.1	22.54–101.88	12.89	2.20		0.04	8.352	OD
		10	*qAD10_130*	8	PHM3736.11	PZA03605.1	125.47–131.38	15.65	2.29		−0.83	9.220	OD
	PH	1	*qPH01_45*	149	PZA00962.1	PZA00939.1	42.32–104.96	4.84	9.75	25.07	2.15	0.631	PD
		6	*qPH06_155*	106	PHM4503.25	PZA02815.25	154.00–160.83	3.05	4.26		1.48	0.142	A
		8	*qPH08_104*	32	PZA01972.14	PZA02566.1	103.35–104.22	3.38	8.35		2.11	0.302	A
		8	*qPH08_130*	67	PZA00460.8	PHM4203.11	126.42–156.31	5.44	7.74		2.05	0.007	A
	EH	1	*qEH01_21*	136	PZA02393.2	PZA00962.1	16.42–42.32	4.88	8.55	26.35	1.68	0.895	PD
		1	*qEH01_220*	189	PZA00664.3	PHM4992.10	216.05–225.87	3.18	6.72		1.59	−0.229	A
		3	*qEH03_50*	48	PZA00581.3	PZA02645.2	46.96–89.25	4.75	6.45		1.63	0.490	PD
		8	*qEH08_130*	67	PZA00460.8	PHM4203.11	126.42–156.31	3.20	4.03		1.20	0.521	PD
	EPO	1	*qEP01_214*	188	PHM759.24	PZA00664.3	213.95–216.05	3.09	3.22	11.13	0.00	0.000	A
		3	*qEP03_153*	31	PZA00667.2	PHM9914.11	152.70–154.23	3.77	7.70		0.00	0.000	A
Low N	GY	1	*qGY01_60*	230	PHM4752.14	PHM574.14	59.27–288.94	3.43	6.27	6.20	0.04	0.026	PD
	AD	2	*qAD02_52*	94	PHM3055.9	PHM4259.5	51.96–186.31	4.64	1.52	18.55	1.16	1.128	D
		2	*qAD02_53*	123	PHM4259.5	PZA01991.3	51.96–221.13	4.68	1.52		1.15	1.152	D
		3	*qAD03_120*	41	PZA00363.7	PZA00707.9	98.45–120.53	5.70	1.30		0.27	0.002	A
		3	*qAD03_158*	180	PZA00186.4	PZA01396.1	157.97–159.08	4.49	1.15		−1.44	1.717	D
		4	*qAD04_172*	52	PHM 2006.57	PZA01905.12	171.17–251.33	6.29	1.52		−0.74	0.366	PD
		4	*qAD04_207*	194	PHM3587.6	PHM14055.6	206.77–208.12	6.92	1.45		0.01	−2.298	OD
		7	*qAD07_82*	90	PZA01933.3	PHM3435.6	80.61–141.80	6.43	1.50		0.05	−2.262	OD
		8	*qAD08_126*	170	PHM4203.11	PZA00766.1	126.42–126.71	4.46	1.26		−1.44	1.757	OD
		10	*qAD10_130*	8	PHM3736.11	PZA03605.1	125.47–131.13	7.73	1.24		0.07	−2.885	OD
	PH	1	*qPH01_21*	137	PZA02393.2	PZA00962.1	16.42–42.32	4.45	6.45	18.76	0.84	−0.081	A
		3	*qPH03_175*	18	PZA00892.5	PZA03735.1	173.26–192.67	4.13	5.32		−0.76	0.494	PD
		9	*qPH09_88*	22	PHM1766.1	PZA03235.1	86.54–107.26	3.21	4.32		0.71	−0.517	PD
		9	*qPH09_86*	32	PZA03235.1	PZA00225.8	76.31–86.54	4.02	4.58		−0.76	−0.050	A
	EH	1	*qEH01_21*	140	PZA02393.2	PZA00962.1	16.42–42.32	3.04	3.34	14.42	0.81	−0.257	PD
		3	*qEH03_155*	26	PHM17210.5	PZA00667.2	154.23–170.91	7.63	10.23		1.41	0.700	PD
		3	*qEH03_155*	33	PZA00667.2	PHM9914.11	152.70–170.91	5.32	6.86		−1.25	0.350	PD
		9	*qEH09_108*	21	PZA00323.3	PHM1766.1	107.26–113.71	3.14	3.39		0.84	−0.713	D
	EPO	3	*qEP03_156*	26	PHM17210.5	PZA00667.2	154.23–170.91	9.01	8.12	17.98	0.01	0.002	PD
		3	*qEP03_155*	33	PZA00667.2	PHM9914.11	152.70–170.91	4.70	4.28		0.00	0.001	PD

Opt, Optimum; Low N- low soil N stress management; GY, Grain yield; AD, anthesis date; ASI, anthesis silking interval; PH, plant height; EH, ear height; EPO, ear position. The italic values refer to the names of identified QTLs.

**TABLE 6:** Genetic characteristics of detected QTLs for GY and associated traits under optimum and low-nitrogen stress in F3 population 3.

Mgmt	Trait	Chr	QTL name	Position (cM)	Left marker	Right marker	Physical position (Mbp)	LOD	PVE (%)	TPVE (%)	Add	Dom	QTL
**F3 pop 3 CML550**×**CML507**													
Opt	GY	9	*qGY09_25*	126	PZB01110.6	sh1.11	17.01–29.66	3.20	3.58	12.15	0.02	−0.003	A
		10	*qGY10_130*	7	PHM3736.11	PZA03603.1	125.47–131.38	3.87	6.21		0.00	0.035	OD
	AD	3	*qAD03_175*	2	PZA01962.12	PZA00892.5	170.91–192.67	3.71	6.09	11.8	0.15	0.003	A
		5	*qAD05_72*	61	PHM13675.17	PZA00261.6	71.08–79.98	3.40	5.07		−0.11	0.115	D
		8	*qAD08_70*	19	PZA01363.2	PZA03012.7	65.14–107.76	4.49	7.10		−0.17	−0.065	PD
	PH	1	*qPH01_210*	153	PZA00664.3	PHM4997.11	6.02–216.05	4.71	5.59	25.81	−1.58	0.298	A
		1	*qPH01_05*	181	PHM4997.11	PZA02129.1	3.83–6.02	3.49	3.87		1.20	0.271	PD
		4	*qPH04_40*	166	PZA03597.1	PZA00541.1	36.33–61.17	3.97	4.32		1.22	−1.050	D
		8	*qPH08_125*	43	PZA01049.1	PZA00770.1	122.03–127.01	4.64	5.31		1.58	0.312	PD
		9	*qPH09_25*	126	PZB01110.6	sh1.11	17.01–29.66	4.70	5.16		1.56	−0.347	PD
	EH	5	*qEH05_165*	95	PZA00148.3	ae1.7	163.41–167.39	3.49	4.67	22.25	−0.71	−0.126	A
		8	*qEH08_125*	43	PZA01049.1	PZA00770.1	122.03–127.01	5.37	7.48		0.82	0.330	PD
		9	*qEH09_108*	123	PHM 1911.173	PZB00544.2	28.74–119.42	5.19	7.62		0.79	−0.520	PD
Low N	GY	1	*qGY01_200*	22	PHM 1968.22	PZA01294.2	63.80–202.86	3.07	5.21	5.12	0.02	−0.014	PD
		10	*qGY10_120*	34	PZA01642.1	PHM15868.56	12.42–120.37	3.45	1.16		−0.33	−0.333	D
	AD	3	*qAD03_175*	0	PZA01962.12	PZA00892.5	170.91–192.67	3.44	5.74	7.45	0.20	−0.174	D
	PH	2	*qPH02_25*	82	PHM4425.25	PZA02378.7	19.52–33.65	3.02	5.00	7.27	−2.95	0.498	A
	EH	2	*qPH02_80*	109	PHM10321.11	PZA01280.2	61.36–143.35	4.07	3.87	13.43	0.57	−0.199	PD
		5	*qPH05_10*	4	PZA01327.1	PZA02367.1	8.62–15.22	3.03	5.14		0.65	1.150	OD
		6	*qPH06_25*	86	PZA02815.25	PHM15961.13	8.62–160.83	4.54	7.18		−0.80	−0.006	A
		8	*qPH08_140*	44	PZB01454.1	PZA00838.2	138.96–151.85	4.52	5.04		−0.71	0.316	PD

Opt, Optimum; Low N- low soil N stress management; GY, Grain yield; AD, anthesis date; PH, plant height; EH, ear height. The italic values refer to the names of identified QTLs.

**TABLE 7:** Genetic characteristics of detected QTLs for GY and associated traits under optimum and low-nitrogen stress in F3 population 4.

Mgmt	Trait	Chr	QTL name	Position (cM)	Left marker	Right marker	Physical position (Mbp)	LOD	PVE (%)	TPVE (%)	Add	Dom	QTL
**F3 pop 4 VL081452**×**VL058589**													
Opt	GY	2	*qGY02_25*	186	PZA01820.1	PZA02264.5	2.55–46.62	3.26	2.14	7.68	0.05	1.243	OD
		10	*qGY10_130*	137	PZA00062.4	PZA00409.17	46.11–130.61	3.49	3.17		−0.01	1.381	OD
	AD	8	*qAD08_25*	139	PZA01196.2	PZA03612.1	24.45–121.52	3.79	10.73	12.64	−0.04	0.422	OD
	EH	2	*qER02_03*	173	PZA00680.3	PHM5535.8	1.00–3.46	6.24	3.05	15.26	0.07	0.763	OD
		2	*qER02_185*	196	PZA02170.1	PHM5060.12	184.40–231.71	6.06	2.87		0.00	0.823	OD
		5	*qER05_55*	194	PZA02207.1	PHM2769.43	51.25–59.62	5.78	3.77		0.01	0.624	OD
		5	*qER05_190*	213	PHM2769.43	PHM7908.25	59.62–191.25	10.17	3.90		0.02	0.782	OD
		6	*qER06_140*	51	PZB01569.7	PZA02478.7	134.48–153.63	3.20	2.72		0.38	−0.369	D
		8	*qER08_30*	160	PZA01196.2	PZA03612.1	24.45–121.52	5.89	3.39		−0.03	0.728	OD
		10	*qER10_80*	10	PHM3309.8	PZA01451.1	7.12–128.41	3.43	3.00		0.30	−0.397	OD
		10	*qER10_130*	135	PZA00062.4	PZA00409.17	46.11–130.61	3.88	1.95		−0.05	0.846	OD
Low N	GY	1	*qGY01_125*	204	PZA02135.2	PZA01254.2	105.20–155.34	3.65	6.42	19.16	0.02	0.005	PD
	AD	1	*qAD01_200*	310	PZA01216.1	PZA03265.3	190.67–203.29	3.91	4.99	35.24	−0.41	−0.109	PD
		2	*qAD02_30*	188	PZA01820.1	PZA02264.5	2.55–46.62	3.65	4.77		0.15	1.752	OD
		7	*qAD07_125*	41	PHM9162.135	PZA02959.14	120.22–133.75	11.05	15.22		0.71	0.077	A
		10	*qAD10_125*	25	PHM3309.8	PZA01451.1	7.12–128.41	3.20	10.51		0.57	0.344	PD
	PH	2	*qPH02_230*	5	PZD00022.5	PZA02727.1	228.51–233.61	3.84	7.10	6.34	1.33	−2.460	OD
	ASI	4	*qASI04_05*	170	PHM3963.33	PHM3301.28	5.54–5.81	4.00	13.58	16.73	−0.03	−0.103	OD

Opt, Optimum; Low N- low soil N stress management; GY, Grain yield; AD, anthesis date; ASI, anthesis silking interval; PH, plant height; EH, ear height. The italic values refer to the names of identified QTLs.

Eleven QTLs were identified for GY under optimum (7) and low soil nitrogen (4) conditions distributed across all chromosomes except chromosome 4, 5, 6, and 8 (Tables 4–8). Across all genotypes and nitrogen regimes, no common QTL was identified. QTLs associated with GY under low nitrogen were detected in chromosomes 1 (population 2, 3, and 4) and 10 (population 3). On the other hand, QTL underlying GY under optimum nitrogen conditions were observed on chromosomes 1 (population 1), 2 (population 4), 8 (population 2), 9 (populations 1 and 3), and 10 (population 4). Under optimum conditions, the total phenotypic variance explained (TPVE) by all QTL was 11.58%, 16.52%, 12.15%, and 7.68% for populations 1, 2, 3, and 4, respectively. TPVE by all QTL under low nitrogen conditions was 6.20% (population 2), 5.12% (population 3) and 19.16% (population 4). Among the QTL detected for GY, we observed additive, dominance, partial dominance and over dominance effect QTL across 4 populations.

**TABLE 8:** Summary of detected QTL for measured traits under optimum and low-nitrogen stress in F3 populations derived from seven elite lines.

Traits	Population	Management	No of QTL	TPVE* (%)
AD	F3pop1CML494×CML550	Optimum	1	8.09
	F3pop2CKL5017×CML536		9	18.89
	F3pop3CML550×CML507		3	11.80
	F3pop4VL081452×VL058589		1	12.64
	F3pop1CML494×CML550	Low N	1	7.70
	F3pop2CKL5017×CML536		9	18.55
	F3pop3CML550×CML507		1	7.45
	F3pop4VL081452×VL058589		4	35.24
ASI	F3pop1CML494×CML550	Low N	3	6.91
	F3pop4VL081452×VL058589		1	16.73
EH	F3pop1CML494×CML550	Optimum	4	23.20
	F3pop2CKL5017×CML536		4	26.35
	F3pop3CML550×CML507		3	22.25
	F3pop4VL081452×VL058589		8	15.26
	F3pop1CML494×CML550	Low N	1	4.87
	F3pop2CKL5017×CML536		4	14.42
	F3pop3CML550×CML507		4	13.43
EPO	F3pop1CML494×CML550	Optimum	2	15.56
	F3pop2CKL5017×CML536		2	11.13
	F3pop1CML494×CML550	Low N	2	11.56
	F3pop2CKL5017×CML536		2	17.98
GY	F3pop1CML494×CML550	Optimum	2	11.50
	F3pop2CKL5017×CML536		2	16.52
	F3pop3CML550×CML507		2	12.15
	F3pop4VL081452×VL058589		2	7.68
	F3pop2CKL5017×CML536	Low N	1	6.20
	F3pop3CML550×CML507		2	5.12
	F3pop4VL081452×VL058589		1	19.16
PH	F3pop1CML494×CML550	Optimum	1	6.01
	F3pop2CKL5017×CML536		4	25.07
	F3pop3CML550×CML507		5	25.81
PH	F3pop1CML494×CML550	Low N	3	9.11
	F3pop2CKL5017×CML536		4	18.76
	F3pop3CML550×CML507		1	7.27
	F3pop4VL081452×VL058589		1	6.34

*TPVE, total phenotypic variance explained; Low N- low soil N stress management; GY, Grain yield; AD, anthesis date; ASI, anthesis silking interval; PH, plant height; EH, ear height; EPO, ear position.

For AD, 26 significant QTLs were identified across the two nitrogen regimes. The QTLs were detected on chromosomes 1, 2, 3, 4, 7, and 10 under low nitrogen and on chromosomes 1, 2, 3, 4, 5, 6, 8, and 9 under optimum nitrogen conditions. No common QTL for the two nitrogen regimes was identified across the studied F3 populations. TPVE by all QTL under optimum conditions was 8.09%, 18.89%, 11.80% and 12.64% for populations 1, 2, 3 and 4, respectively. Under low nitrogen conditions, TPVE by all QTL was 7.70% (population 1), 18.55% (population 2), 7.45% (population 3) and 35.24% (population 4). The nature of the QTLs classified a few as additive, dominant, partial dominant and overdominance groups.

Four QTLs were identified for ASI under low nitrogen conditions. These were detected on chromosomes 1, 3, 4, and 6. As in the case of GY and AD, no common QTL was identified. TPVE by all QTLs under low nitrogen conditions was 6.91% (population 1) and 16.73% (population 4).

For EH, 24 significant QTLs were detected with 9 of those under low soil nitrogen conditions. The QTLs underlying EH under low soil nitrogen conditions were detected in chromosomes 1, 2, 3, 5, 6, 7, 8, and 9. Under optimum conditions, the QTLs were found in all 10 maize chromosomes except for chromosome 7. Common QTL for the two nitrogen regimes was not identified. TPVE by all QTLs under optimum conditions was 23.20%, 23.35%, 22.25% and 15.26% for populations 1, 2, 3, and 4, respectively. Under low soil nitrogen conditions, TPVE by all QTL was 4.87% (population 1), 14.42% (population 2), and 13.43% (population 3).

Significant QTLs underlying EPO were recorded as 7 under both nitrogen regimes. One QTL (Chromosome 6: *qEPO6_157*) was found in both low and optimum nitrogen conditions. Under low soil nitrogen management, QTLs were detected in chromosomes 3, 4, and 6. On the other hand, chromosomes 1, 3, 6, and 8 housed QTL underlying EPO under optimum conditions. Under optimum conditions, the TPVE by all QTL was 15.56%, and 11.13% for populations 1 and 2, respectively. TPVE by all QTLs under low nitrogen conditions was 11.56% (population 1) and 17.98% (population 2).

For PH, 19 significant QTLs were identified across the studied genotypes and nitrogen regimes. Nine of those were identified under low nitrogen conditions in chromosomes 1, 2, 3, 6, 7, and 9. Under optimum conditions, significant QTLs were detected in chromosomes 1, 4, 6, 8, and 9. No common QTL for the two nitrogen regimes was identified across the studied F3 populations. TPVE by all QTLs under optimum conditions was 6.00%, 25.07%, and 25.81% for populations 1, 2, and 3, respectively. Under low nitrogen conditions, TPVE by all QTL was 9.11% (population 1), 18.76% (population 2), 7.27% (population 3) and 6.34% (population 4).

### 3.4 Overlapping QTL for each trait evaluated under low and optimum nitrogen conditions

The identification of common QTLs is crucial in targeting markers that can be used in breeding for improved nitrogen stress tolerance and NUE. This study identified several common QTLs across the studied F3 populations and nitrogen regimes (Tables 4–8). Under optimum conditions, one QTL (*qAD03_158*) for AD was overlapping with one QTL under low soil nitrogen conditions on chromosome 3 (157.97–159.08 Mbp). For ASI and PH, no common QTL was identified across the two nitrogen regimes. Under low soil nitrogen conditions, one QTL for EH, *qEH01_21* (16.42–42.32 Mbp), was overlapping with two QTLs under optimum conditions (from 20.99 to 21.87 Mbp and 16.42 to 42.32 Mbp) on chromosome 1. Similarly, under low nitrogen, the second QTL for EH (*qEH09_108*: 107.26 to 113.71 Mbp) overlapped with one QTL under optimum conditions (28.74–119.42 Mbp) on chromosome 9. One common QTL for EPO, *qEP06_157*, was found under both low and optimum nitrogen conditions (156.17–159.55 Mbp and 156.17 to 159.55 Mbp) on chromosome 6. Under optimum conditions, one QTL for GY (*qGY10_130*) was found on chromosome 10 in both population 3 (125.47–131.38 Mbp) and population 4 (46.11–130.61 Mbp). A similar observation was made for one QTL underlying EH (*qEH09_108*) which was seen on chromosome 9 under optimum nitrogen conditions in population 3 (28.74–119.42 Mbp) and low nitrogen conditions in population 2 (107.26–113.71 Mbp). This study found no QTL correspondence among the studied traits across the nitrogen regimes.

### 3.5 Genomic prediction correlations for traits evaluated under low and optimum nitrogen conditions

**FIGURE 3:**
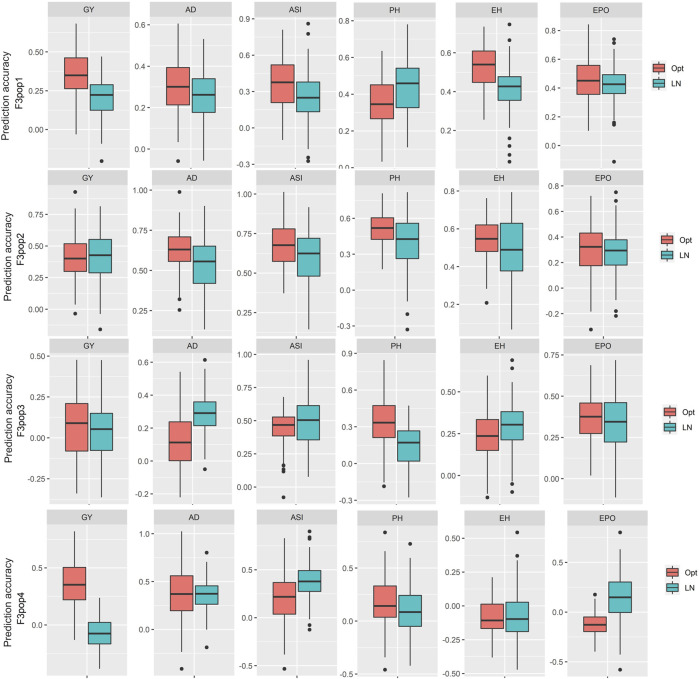
Distribution of the five-fold cross-validated genomic prediction correlations for grain yield (GY), anthesis date (AD), anthesis-silking interval (ASI), plant height (PH), ear height (EH), and ear position (EPO) evaluated in multiple environments under optimum and low soil N management conditions in four F_3_ populations.

To assess the potential breeding value of GY and other agronomic traits under optimum and low soil N management, GS analysis was performed on all four populations using NUE-associated traits and genotypic markers with RR-BLUP. The RR-BLUP prediction correlations were 0.35, 0.41, 0.06, and 0.35 in populations 1, 2, 3, and 4, respectively (Figure 3; Table 9) under optimum, whereas under low soil N management, the correlations were reduced to 0.21, 0.41, 0.04, and −0.02 in population 1, 2, 3, and 4, respectively. For AD, prediction correlations were 0.31, 0.63, 0.12, and 0.37 under optimum and 0.26, 0.43, 0.29, and 0.36 under low soil N management, respectively (Figure 3; Table 9). For PH, prediction correlations were 0.35, 0.52, 0.34, and 0.18 under optimum, and 0.44, 0.40, 0.15, and 0.10 under low soil N management in populations 1, 2, 3, and 4, respectively. Overall, the prediction correlations were higher in population 2 and lower in population 4 for all the traits under both optimum and low soil N management.

**TABLE 9:** The prediction accuracy for grain yield and other traits evaluated under optimum and low-nitrogen stress conditions for four F_3_ populations derived from seven elite lines.

Traits	GY	AD	ASI	PH	EH	EPO
Optimum						
F3pop1CML494×CML550	0.35	0.31	0.37	0.35	0.53	0.45
F3pop2CKL5017×CML536	0.41	0.63	0.68	0.52	0.54	0.29
F3pop3CML550×CML507	0.06	0.12	0.44	0.34	0.24	0.37
F3pop4VL081452×VL058589	0.35	0.37	0.20	0.18	−0.09	−0.11
Low N						
F3pop1CML494×CML550	0.20	0.26	0.25	0.44	0.42	0.42
F3pop2CKL5017×CML536	0.41	0.53	0.59	0.40	0.49	0.28
F3pop3CML550×CML507	0.04	0.29	0.50	0.15	0.29	0.35
F3pop4VL081452×VL058589	−0.07	0.36	0.38	0.09	−0.07	0.14

Low N- low soil N stress management; GY, Grain yield; AD, anthesis date; ASI, anthesis silking interval; PH, plant height; EH, ear height; EPO, ear position.

## 4 Discussion

### 4.1 Impact of low nitrogen stress on GY and other associated traits

The role of nitrogen stress in yield reduction and overall crop development cannot be overstated. An in-depth understanding of GY and other related agronomic traits is critical for the evidence-based development of varieties that are tolerant to low soil nitrogen stress (Derera et al., 2008). However, phenotypic characterization of such complex traits is very challenging due to unpredictable environmental and edaphic conditions (Liu et al., 2010). Importantly, selection through morphological characterization under nitrogen stress conditions is very inconsistent and will not give correct phenotypic data thereby derailing progress in maize breeding schemes. In contrast, the integration of precise genomic tools coupled with conventional breeding can accelerate the development of nitrogen-stress adaptive cultivars with high yields. In this research, two nitrogen regimes (i.e., low, and optimum nitrogen) served as the basis for the identification of QTLs related to GY and other agronomic traits (i.e., AD, ASI, PH, EH, and EPO) and their cross-cutting heritabilities in four F_3_ maize populations.

The target traits measured followed a normal distribution indicating that the studied traits are quantitative in nature and the tested F_3_ populations are stable and suitable for QTL mapping. The mean GY reduction in this study was 69.82% under low nitrogen stress. This is very close to the 71% yield reduction recorded in the study by Ertiro T. B. et al. (2020). Yield reduction under low soil nitrogen stress demonstrates the importance of nitrogen in the growth and development of maize (Ertiro T. B. et al., 2020). Numerous studies have linked a reduction in yield under low-N conditions to decreased kernel number due to abortion (Bänziger et al., 1997; Agrama et al., 1999; Ribaut et al., 2007). Low soil nitrogen environments decrease the number of kernels and ears per plant, decrease the chlorophyll concentration in the ear leaf, and lowers PH by roughly half (Ribaut et al., 2007).

Genotypic and genotype × environment interaction effects were significant for GY across the tested nitrogen regimes. Generally, across the studied environments, the most responsive trait was AD. Our results showed that low nitrogen stress extends the AD in maize. Furthermore, low nitrogen stress increased genetic variance for AD across the studied genotypes. This can be viewed as an indication of the adaptive nature of this secondary trait.

Across the four F_3_ populations, there was a consistent moderate to low heritability on all the studied traits across the two nitrogen regimes. For all traits, heritability was shown to decline under low nitrogen stress. The heritabilities of almost all the studied traits were moderate to low. Similar trends were also reported in earlier studies with DH populations (Ertiro et al., 2020b; Ertiro et al., 2022) and association panel (Ertiro et al., 2020a) which were evaluated under optimum and low soil N conditions. Despite this, the heritabilities were significant enough to facilitate indirect selection for increased GY under low nitrogen stress. By comparison, PH had low heritabilities under both low and optimum nitrogen conditions.

The pairwise correlation showed a strong negative correlation for GY with AD and ASI across the studied nitrogen regimes. However, other agronomic traits showed positive correlations with GY. These include PH, EH and EPO under both low and optimum soil nitrogen conditions. Similar correlation trends were also reported by earlier studies with maize hybrid and inbred line trials under low soil nitrogen conditions (Ertiro T. B. et al., 2020; Ertiro et al., 2022). PH, EH and EPO had a positive correlation with GY suggesting that improvement of these traits lead to improved maize varieties with high-yielding potentials. Furthermore, these correlations suggest the high odds of tightly linked loci controlling low-N tolerance through the coordinated expression of loci controlling these traits.

### 4.2 QTL mapping under low and optimum nitrogen conditions

NUE traits are highly complex, the advent of high-density marker data has made it feasible to dissect their underlying genetics. The detection of QTLs underlying GY and other associated traits under different nitrogen regimes is integral for scaling breeding initiatives targeting the development of low nitrogen tolerant maize varieties. A total of 91 significant QTLs were identified in this study for six traits under low and optimum nitrogen management (Tables 5, 6, 7, and 8). The disparity in the number of QTLs under optimum (52) and low (39) indicates the effect of genetic variance across the environments. Some of these were found across management conditions and populations. Across the two nitrogen regimes, chromosomes 1 (16) and 8 (14) had the highest number of QTLs whilst chromosomes 7 (5) and 5 (6) had the lowest. Chromosomes with a high number of QTLs can be targeted for future research studies focusing on improving GY under low nitrogen stress. Proportional phenotypic variance for the QTLs ranged from 0.7% to 15.22%, with an average of 5.05%.

Population 4 had the highest number of QTLs (36). This is a testament to the diversity within the studied F_3_ populations across nitrogen management options. AD (26) and EH (24) had the highest number of QTLs whilst ASI (11) had the lowest. This is consistent with another study (Ertiro et al., 2020c) which found the highest QTL number as those underlying AD. In our study, this was consistent with the observed high genetic variance for AD and EH across the two nitrogen regimes.

Marker-assisted selection for GY improvement relies on the successful identification of QTLs with moderate to high effects. For GY, 11 QTLs were detected under both optimum (7) and low (4) nitrogen conditions. This is inconsistent with Ertiro et al., 2020c findings which recorded more QTLs under low nitrogen management than under optimum conditions. One QTL for GY (*qGY10_130*) was found under optimum conditions on chromosome 10 in both population 3 (125.47–131.38 Mbp) and population 4 (46.11–130.61 Mbp). A study by Ertiro et al., 2020c also found QTLs underlying GY under different nitrogen regimes on chromosome 10. No common QTL for GY was identified across the studied F_3_ populations. Ertiro et al. (2020a) also observed similar results, i.e., no common QTLs observed for GY. Noteworthy, the use of a different number of populations, locations and markers in future studies can give rise to a different outcome. In this study, some common QTLs were identified for AD, EH and EPO. The identification of common QTLs justifies the magnitude of correlation across the two nitrogen regimes. Common QTLs can also be used for indirect selection. Similar to the results of Ertiro et al., 2020c, QTL correspondences detected between the nitrogen regimes for the studied traits were not similar across F_3_ populations and this points to the genetic-background-specific nature of these QTLs.

The distinct genetic controls of the expression of the phenotypes reported in this study and related studies (Bänziger et al., 1999; Ribaut et al., 2007) suggest that selection for low nitrogen stress tolerance will be more effective under low nitrogen management. These findings unravel the most promising genomic regions for MAS to increase maize tolerance to low nitrogen stress. Further multi-environment trials with larger population sizes are necessary to validate the stability of the recorded QTLs and utilize them to identify underlying causal genes.

To achieve higher genetic gain, complex traits in maize breeding can be improved by integrating genomic, bioinformatic, and statistical tools into breeding programs (Beyene et al., 2019; Beyene et al., 2021). Rapid technological advancements have made available cheaper genotyping tools, while rapid progress in the field of big data and bioinformatics has led to the development of user-friendly software tools which can handle complex statistical models and facilitates breeders to apply them to improve complex traits on a more routine basis. GS, which predicts the breeding values by employing genome-wide markers is proving to be effective in improving complex traits that are controlled by multiple minor effect genes. For instance, RR-BLUP or G-BLUP is now very commonly used to predict several complex traits in maize (Guo et al., 2012; Hao et al., 2019; Beyene et al., 2021). Several NUE traits like chlorophyll index, chlorophyll fluorescence and leaf N content were also selected by genomic prediction before integrating into NUE breeding programs in ryegrass (Zhao et al., 2020). In the present study, GS was performed on GY and other agronomic traits evaluated under optimum and low soil N stress conditions. The average genomic prediction correlations ranged from 0.07 to 0.41 under optimum and −0.07 to 0.41 under low soil N stress conditions. The higher values of prediction correlations are comparable to earlier studies with a diversity panel evaluated under optimum and low soil N stress conditions (Ertiro et al., 2020c). The observed prediction accuracies for other agronomic traits are comparable to earlier studies reported under different stresses in maize (Zhang et al., 2015; Yuan et al., 2019).

In population 3, we found low accuracy for GY under optimum condition, which could be due to its small range of variability within the population as well as low heritability (Table 2, Figure 1). We observed a wide range of prediction correlations for same trait among different populations and management. This could be due to their differences in sample size, genetic variance, trait heritability, changes in population structure and linkage disequilibrium estimates. In some populations, we observed negative prediction correlations like GY for pop 4 under low soil N stress conditions (Figure 3; Table 9). Opposite linkage phases between markers and major-effect QTLs in the population may be the reason for negative correlations. In addition, the four F_3_ populations used in this study were developed by using tolerant x tolerant crosses for low soil N stress, which may be another reason why we observed low prediction correlations (as most of the causal factors for these traits might have been fixed on both the parental lines). As a result, variation is low for the traits which is evident with low to moderate heritability estimates (Table 2). Nevertheless, the prediction correlation followed a consistent pattern for all traits under both optimum and low N conditions. Traits with high prediction correlations also tend to have relatively high heritability estimates. In GS, the less complex trait AD and PH had higher accuracy compared to GY, which is consistent with the nature of trait complexity (Zhang et al., 2017a; Yuan et al., 2019). For simple inherited traits that are positively correlated with GY, prediction correlations are moderate to high, which clearly supports the usefulness of GS for their improvement under either optimum or low soil N stress conditions.

Overall, the linkage mapping studies with our F_3_ populations revealed several QTL with minor to moderate effects for GY and other agronomic traits under optimum and low N management. It is difficult to capture multiple QTL with small to moderate effects for selection and their environmental and genotypic specific expressions make it even more difficult to improve these traits only through traditional breeding or a few QTL based MAS. However, discovery of genomic regions through linkage mapping will continue to be vital to understanding the genetic basis of these traits. On the other hand, genome-wide selection is critical in improving quantitative traits. The phenotypic selection efficiency per cycle is measured as *h* (square root of heritability) and the value of marker-based prediction accuracy close to *h* indicates the selection response based on markers and based on phenotypes are near equal (Dekkers, 2007; Lorenzana and Bernardo, 2009). The prediction accuracies observed for GY and other traits was ≥1/2*h* indicating that the response to marker-based selection would be at least 50% of the response to phenotypic selection for agronomic traits. With the possibility of making three cycles per year by using marker-based selection, the selection response will be −1.5 times the gain from one cycle of phenotypic selection. These results indicate that genome-wide selection would be more efficient in terms of genetic gain per year. However, one must be cautious as we observed prediction accuracy of <0.10 for some traits in some populations, where GS has no additional advantage over phenotypic selection.

## 5 Conclusion

Nitrogen-depleted soils are a major factor behind low maize productivity in smallholder farming systems in SSA. Genomics-based plant breeding techniques such as QTL mapping and GS can provide useful information for scaling the development of low nitrogen tolerant maize varieties. Here, we sought to identify the genomic regions associated with GY and related traits under optimum and low soil nitrogen conditions in F_3_ maize populations grown in Kenya and Zimbabwe. Our analysis found a total of 91 QTLs underlying GY, ASI, AD, EH, EPO, and PH. However, no common QTL for GY was identified across the studied nitrogen regimes. The validation of these QTLs in the same F_3_ populations is needed to guarantee the success of future marker-assisted selections. Identification of many QTL with minor effects indicates rather QTL mapping on these traits, GS is more appropriate for their improvement. Genomic prediction correlations were low to moderate. However, by considering the possibility to have three cycles per year with marker-based selection, integration of GS in a low N tolerant breeding program will be more efficient to improve GY under low soil N conditions.

## Data Availability

The original contributions presented in the study are included in the article/Supplementary Material. This data can also be accessed here: https://zenodo.org/records/10021760.
